# Attitude toward physical activity after total hip or knee replacement: A cross-sectional survey study of Dutch and Norwegian patients

**DOI:** 10.1371/journal.pone.0325746

**Published:** 2026-01-23

**Authors:** Inge van den Akker-Scheek, Martin Stevens, Astrid J. De Vries, Anne Marie Fenstad, Ove Nord Furnes, Håvard Østerås, Bregje E. Raap – van Sleuwen, Katja Saris, Geranda E.C. Slager, Monica Unsgaard-Tøndel, Odd Magne Hals, Ann-Katrin Stensdotter

**Affiliations:** 1 Department of Orthopaedics, University of Groningen, University Medical Center Groningen, Groningen, The Netherlands; 2 Department of Orthopaedic Surgery, Martini hospital, Groningen, The Netherlands; 3 The Norwegian Arthroplasty Register, Department of Orthopedic Surgery, Haukeland University Hospital, Bergen, Norway; 4 Department of Clinical Medicine, University of Bergen, Bergen, Norway; 5 Faculty of Medicine and Health Sciences, Dept. of Neuromedicine and Movement Science Norwegian University of Science and Technology, Trondheim, Norway; 6 Department of Orthopaedic Surgery/Research Department, Bernhoven, Uden, The Netherlands; 7 Department of Orthopaedics, Radboud University Medical Center, Nijmegen, The Netherlands; 8 Department of Physical Therapy, School of Health Care Studies, Hanze University of Applied Sciences, Groningen, The Netherlands; Leiden University Medical Center, NETHERLANDS, KINGDOM OF THE

## Abstract

**Objective:**

To gain insight into the attitude towards physical activity (PA) of Norwegian and Dutch patients after hip or knee replacement, possible differences in attitude between the countries and which factors explain a more positive or negative attitude.

**Methods:**

A cross-sectional survey study investigating attitude toward PA 6–12 months after hip or knee replacement was conducted in Norway and the Netherlands. Patients received a questionnaire consisting of three parts: demography, received health service, and the survey about attitude toward PA consisting of 32 statements divided into four domains – quality of life, level of PA, function, and kinesiophobia. Attitude was scored on a 4-graded Likert scale. A high score indicated a positive attitude toward PA. Survey responses from the Norwegian and Dutch cohorts were compared using one-way ANOVA for group comparison. To identify factors explaining the outcome of the attitude survey, stepwise regressions were used.

**Results:**

Data from 732 Norwegian patients and 575 Dutch patients was included in the analyses. Overall, the characteristics of the two cohorts were quite similar, although a significantly greater number in the Norwegian cohort had participated in “prehab”, while the participation in “rehab” was similar. In all four domains the attitude scores were generally high, indicating positive attitudes, with Norwegian patients scoring significantly higher. Higher participation in sports was the strongest explaining factor for a more positive attitude toward PA. Older age was the strongest independent variable explaining a less positive attitude towards PA.

**Conclusion:**

Patients seem to have an overall positive attitude towards PA after hip or knee replacement. Norwegian patients had a more positive attitude compared to Dutch patients, which could be the result of more formalized and extensive education of the benefits of PA as part of the “prehab” in Norway.

## Introduction

Total Hip and Total Knee Arthroplasty (THA/TKA) are considered the preferred treatment for end-stage osteoarthritis (OA) [1,2]. In 2019, the Organization for Economic Co-operation and Development (OECD) reported 267,5 THA and 117,4 TKA cases per 100,000 population in Norway (approx. 5 million inhabitants), and the Netherlands reported 253,9 THR and 155,3 TKA per 100,000 (approx. 17 million inhabitants) [3]. THA and TKA are cost-effective, pain relieving and improves the ability to stay physically active [4]. After THA and TKA it is of the utmost importance that patients have a physically active lifestyle, not only from a general health and fitness perspective but also because physical activity (PA) benefits the prosthetic joint [5].

Regular PA is recognized as a key factor of lifestyle behavior, enhancing general health and fitness [6]. There is overwhelming evidence stating that PA prevents several chronic diseases and decreases mortality [7,8]. PA also improves fitness, which is an essential contributor to retain function and improve the ability to keep up activities of daily living (ADL), independence, and participation with advancing age [9]. Being physically active after THA or TKA is particularly important for reducing fall risk and maintaining bone density which is essential for improved fixation and reduced risk of loosening of the prosthesis [5]. Recognition of the importance of PA has led to international recommendations for health-enhancing PA issued by the WHO and national health institutes [6]. These recommendations comprise at least 150 minutes per week of moderate PA, such as walking and cycling, spread over several days. Longer, more frequent and/or more intensive exercise provide additional benefits in terms of fitness and health. In addition to these 150 minutes per week of moderate PA, it is recommended to do muscle strengthening activities at least twice a week (such as climbing stairs, standing up from a sitting position and strength training), and for the elderly combined with balance exercises. Moreover, it is recommended to limit sedentary time and replace it with activity of any intensity.

From former studies it is known that only a limited number of people comply to the PA guidelines, and that this is even worse in the group of patients after THA and TKA, despite the known benefits [10]. Patients often report pain and loss of function due to osteoarthritis as reasons for being physically inactive, but with a total joint prosthesis their PA behavior does not seem to change [10,11]. A systematic review of thirteen papers including 282 patients who had undergone THA or TKA revealed that patients generally wish to return to pre-pathological level of PA, however showing limited interest in actually undertaking PA either for pleasure or health gains [12]. To improve education and support for these patients to facilitate change, it is important to have insight into their attitude towards PA behavior after THA and TKA, however, this information is lacking in current literature. Moreover, insight into the attitude of patients from different countries can provide additional insights as culture, lifestyle, and health care systems among other things will vary. Within Europe, Scandinavia can be considered frontrunner in conservative OA care, providing education and PA promotion programs [13–15]. In Norway, collaboration between the physiotherapist and the general practitioner in primary health care is emphasized for coherent OA care [14,16].

Against that background, the objective of this study was to gain insight into the attitude towards PA of Norwegian and Dutch patients 6–12 months after hip or knee replacement, possible differences in attitude between the countries and which factors that may contribute to explain the attitude toward PA.

## Methods

### Design

A cross-sectional survey study was used for investigating attitude toward PA 6–12 months after THA or TKA. This study was a part of an ERASMUS project “*PAIR: Physical activity after hip or knee replacement*”, involving several European countries [17]. In the present study, data from Norway and the Netherlands were used. The choice of data selection was motivated by the equal and sufficiently high number of survey respondents regarding calculations of statistical power, which was not reached by the other participating countries [17].

### Data collection

Data was collected from patients aged 18 years or older who had primary osteoarthritis in the hip or knee and had undergone THA or TKA. In Norway, the Norwegian Arthroplasty Register (NAR) manages the data collection nationwide from patients living in Norway [18]. On 28-06-2021, questionnaires were distributed by mail with response envelopes to the cohort that had undergone a THA or TKA 6–12 months prior. A stratified selection secured representation for equal male/ female distribution, age and geographical area. A reminder was sent on 09-09-2021. In the Netherlands, the National Orthopedic Prosthesis Register (LROI) does not contain contact information of the patients. Therefore, another data collection approach was used; data collection was managed by University Medical Center Groningen (UMCG) and collected from four hospitals based on geographical distribution and type (one university hospital, three regional (teaching) hospitals). Questionnaires including response envelopes were anonymously handed out during the inclusion period (01-11-2021 until 01-08-2022) to all patients who had THA or TKA and came for their 6- or 12-months control visit to the outpatient clinic of one of the four participating hospitals. No reminders were sent. The sample size was calculated based on the number of primary THA or TKA surgeries per year in each country. Calculation was done to secure that the sample was large enough to represent the population within a given confidence interval. The confidence level was set at 95%. The confidence interval was estimated 87.4 to 92.6 (≈ 5). Considering a non-response of 40%, enrolment of 437 Norwegian and 455 Dutch patients was needed.

### Questionnaire

As there was no existing questionnaire to measure attitude, two authors (AKS and OMH) made a first draft of a study-specific questionnaire based on literature [19–22] and all partners in the PAIR project gave feedback. Variations were discussed to accommodate for differences between the different partner countries. After a final version was agreed upon, the questionnaire was translated into the language of each country, and then back translated. The final version was validated for context and content validity in each country by a pilot survey with approximately 10 patients who gave their feedback. The questionnaires were adjusted accordingly for each separate country. The Norwegian and Dutch versions were used in the current study.

The questionnaire consisted of three parts: the first part was patient characteristics (age, education level, occupation, other diagnoses, walking aids etc.). Factors such as age, other diagnoses and walking aids can have a decisive impact on attitude and were therefore considered important to assess. The second part was about received health service and included questions about time since surgery and if participants had participated in any rehabilitation program before/after surgery and received information on PA, which all could have influenced their attitude. The third part comprising the actual survey about attitude toward PA, consisting of 32 statements divided between four domains: quality of life (8 statements), level of physical activity (11 statements), function (4 statements) and kinesiophobia (9 statements). Attitude was scored according to a 4-graded Likert scale ranging from strongly disagree to strongly agree. A high score indicated a positive attitude toward PA. Items with negative statements were recoded with inverted scores (S1 File).

### Ethics and GDPR

The study was executed in accordance with the Helsinki Declaration. Norwegian participants signed an informed consent, and an ID connected the patient to the data. The ID key was kept by NAR not available to the researchers. In the Netherlands patients were informed that return of the (anonymous) questionnaire was considered as consent to participate. The responses were manually entered into the GDPR approved WebCRF database at Norwegian University of Science and Technology (NTNU) [23] where the UMCG was given an account. The UMCG and NTNU signed a data processor agreement allowing NTNU to process data on the behalf of the data controller. Each partner retained the rights to their own data. A general ethical approval for the transnational survey was granted for NTNU (REK 244244/ 25.08.2021) and a local approval for the Netherlands was granted at UMCG (METc 220/530).

### Statistical analysis

The statistical analyses were conducted using SPSS (IBM, Armonk, NY) version 29. Background characteristics were compared between groups with Pearson chi-square for categorical variables (Table 1). Survey responses from the Norwegian and Dutch cohorts were compared using one-way ANOVA. To identify factors predicting attitudes in the survey, stepwise regressions were performed for the full dataset and separately for each cohort. Variables were entered into the stepwise regression model based on an inclusion criterion of F <=.050 and removed if F >=.100. The final models (for the full dataset, for the Dutch and for the Norwegian cohort respectively) including all variables meeting these criteria, were reported. The number of models for each regression varied between one and four, with the final model including the number total number of entered independent variables explaining R^2^. The regression analysis for the Norwegian cohort was adapted from a previously submitted manuscript in Norwegian language with a related but distinct research objective. Pearson correlation analyses between background factors were performed separately for each country to assess potential collinearity risks in the regression models (S2 Table). To minimize collinearity and enhance interpretability, the background factors were categorized into four domains (demography, lifestyle, health, and health service (S3 Table)). Similarly, survey responses were grouped into four domains corresponding to part three of the survey (quality of life, level of PA, function, and kinesiophobia (S1 File)). Collinearity diagnostics were performed using a tolerance threshold of 0.04. The significance level was set at p < .001.

**Table 1:** Demographics and background information on patients who undergone total hip or knee replacement in the time-period between 1.7.2020-31.12.2020.

Variable	Norway n = 732		The Netherlands n = 575		Group difference
	Percent	Frequency	Percent	Frequency	p-value
Male/female	40.8/59.2	276/400^a^	39.9/60.1	225/340^a^	.516
Married/living together	71.6	524	70.4	393	.376
Age (years):					.039
< 51	2	14	3.6	19	
51-60	15.3	106	15.5	80	
61-70	36.6	253	31.8	164	
71-80	36.5	252	35.9	185	
>80	9.5	66	13.2	69	
Height (cm):					.333
< 161	13.9	98	14.2	80	
161-170	35.6	252	35.7	201	
171-180	29.3	207	28.8	162	
> 180	21.2	150	21.3	120	
Weight (kg):					.248
< 66	13.6	97	24,7	75	
66-70	11.6	83	12.4	71	
71-75	12.5	89	11.7	67	
76-80	10.4	74	13.8	79	
81-85	16.0	114	14.7	84	
86-90	10.7	76	11.5	66	
>91	25.2	180	22.8	131	
Education:					<.001
Grammar school	17.1	121	42.3	224	
College	44.3	324	48.3	256	
Bachelor’s degree	23.2	170	6.4	34	
Master’s degree or higher	12.7	93	3.0	16	
Work:					<.001
Not working	43.0	315	54.8	285	
Office work	21.4	157	13.8	72	
Light physical	25.5	187	20.0	104	
Heavy physical	6.7	49	11.3	59	
Exercise:					<.001
None	21.0	154	32.5	175	
Leisure/ sporadic	29.1	213	25.8	139	
Moderate/ regular	36.1	264	39.8	214	
Intense/ competitive	10.2	75	1.9	10	
Smoking:					.167
Never	48.2	353	43.7	242	
Stopped	42.1	308	48.0	266	
<10/ day	4.8	35	5.2	29	
>10/ day	2.0	15	3.1	17	
Knee prosthesis/ hip prosthesis	47.6/ 52.4	340/374	45.3/ 54.7	236/ 285	.419
Several prostheses	4.6	34	10.1	58	<.001
Comorbidity diagnosis:					.043
None	13.5	99	17.9	103	
One diagnosis	78.1	572	70.8	407	
Several diagnoses	8.3	61	11.3	65	
Walking aids:					<.001
None	86.9	604	74.2	409	
Cane	4.0	28	10.2	56	
Crutches	7.3	51	4.0	22	
Walker	1.7	12	11.6	64	
Participation in prehab program	43.8	239	16.7	90	<.001
Participation in rehab program	70.0	521	69.4	381	.002
Participation frequency* means (SD)	4.56 (2.2)		3.88 (1.8)		<.001
Information about physical activity	98.5	721	96.3	554	<.001

*Total frequency of participation in prehab + rehab, with participation score ‘never’ = 1, ‘weekly for <1 month’ = 2, ‘weekly for 1-2 month’ = 3 and ‘weekly for >2 months’ = 4, resulting in a total score ranging from 2–8.

Missing: range 0.3–6.3, mean 3.4%

^a^not all have filled out gender

## Results

In total, data of 732 Norwegian patients and data of 575 Dutch patients was included in the analyses. The results for the background data as well as the response to the survey on attitudes toward physical activity had some randomly distributed missing data as noted in the tables below.

### Background variables

Table 1 shows the demographic characteristics and report group differences between the Norwegian and Dutch cohorts. There was some randomly missing data as some questions were omitted by some respondents (percent missing range 0.3–6.3, mean 3.4). The proportion male/ female participation was similar between cohorts, as were distribution across categories for age, weight, and height Social factors revealed differences in the work situation between cohorts with a larger proportion not working, less doing office work and more involved in heavy physical work in the Dutch cohort. For education, the Norwegian cohort had a greater proportion of participants with higher education and the proportion of participants with only grammar school was greater for the Dutch cohort. Lifestyle differed significantly where a higher number in the Norwegian cohort were engaged in intensive or competitive sports. In contrast, a greater number in the Dutch cohort responded that they were not sports active. Smoking habits were similar between cohorts. A significantly greater proportion in the Norwegian cohort had participated in “prehab”, i.e., an exercise program before surgery, while the participation in rehab, exercise after surgery, was similar. The general training frequency was however higher in the Norwegian cohort. The proportion of patients reporting that they have received information about physical activity was high in both cohorts, but significantly higher for Norway. The proportion of THA and TKA were similar, but there was a significantly greater number having several previous prostheses in the Dutch cohort. A higher portion in the Dutch cohort used walking aids, while there were no groups differences regarding the number of diagnoses.

### Survey responses for attitudes toward PA

Mean attitude scores were generally high for both cohorts, but there were still some differences (Table 2a–2d). There was some randomly missing data as some questions were omitted by some respondents; on average this was less than 8%. For quality of life (Table 2a), missing ranged between 1.5–10.5% (mean 5.7%). The highest number of missing was found in level of physical activity (Table 2b, range 5.1–8.2%, mean 7.1%). Missing for function (Table 2c), ranged between 3.3–5.3% (mean 4.2%). The lowest number of missing data was found for kinesiophobia (Table 2d) ranging between 2.9–4.4% (mean 3.6%). For the domain *Quality of life* (Table 2a), scores were significantly higher for the Norwegian cohort with mean sum score 28.87 (SD 3.974) compared to the Dutch cohort 25.12 (SD 8.258) (F_1:1296_ = 117.6, p < .001). Significant differences were found for all statements except the last two and negative statements on PA being bad and friends and family’s disapproval. For the domain *Physical activity* (Table 2b), a higher mean sum score was found for the Norwegian cohort 31.99 (SD 6.322) than the Dutch cohort 28.40 (SD 9.531) (F _1:1306_ = 66.7, p < .001). The Norwegian cohort had significantly higher scores for physical demands in daily life and family support, while the Dutch cohort scored higher on endurance training. Moreover, higher scores for the Norwegian cohort were seen on the statements about practicing exercises, ability to perform physical activity, and increasing and not reducing the level of physical activity after surgery. For the domain on *Function* (Table 2c), the Norwegian cohort had a higher mean sum score 14.19 (SD 2.574) than the Dutch cohort 12.85 (SD 3.562) and scored significantly higher on all statements (F = 62.5_1:1306_, p < .001). The domain *Kinesiophobia* (Table 2d), was also scored higher for the Norwegian cohort 28.84 (SD 4.407) than for the Dutch cohort 26.76 (SD 6.061) (F = 51.3_1:1306_, p < .001). Partial scores were significantly higher for the Norwegian cohort for all statements but for “pain stopping activity” there was no difference between cohorts.

**Table 2:** a. Survey responses about attitudes toward physical activity – domain “quality of life”. b. Survey responses about attitudes toward physical activity – domain “level of physical activity”. c. Survey responses about attitudes toward physical activity – domain “function”. 2d. Survey responses about attitudes toward physical activity – domain “kinesiophobia”.

Variable	Norway n = 732		Netherlands n = 575		Groups differences	
	Means [SD]	99.9% CI	Means [SD]	99.9% CI	DF/ F values	p-values
a. Survey responses about attitudes toward physical activity - domain “qulaity of life”						
Sum score: quality of life	28.87 [3.974]	28.38, 29.35	25.12 [8.258]	23.98, 26.26	1:1296/ 117.6	<.001
25. Physical activity is important for my fitness	3.75 [.499]	3.69, 3.81	3.58 [.624]	3.49, 3.67	1:1282/ 29.8	<.001
26. Physical activity is important for my health	3.75 [.486]	3.69, 3.81	3.38 [.890]	3.26, 3.51	1:1265/ 87.6	<.001
27. Physical activity is important for my social life and lifestyle	3.58 [.600]	3.51, 3.65	3.18 [.876]	3.06, 3.31	1:1248/ 90.3	<.001
28. I enjoy being physically active	3.52 [.623]	3.44, 3.60	3.13 [.866]	3.00, 3.25	1:1246/ 86.0	<.001
29. I do not have time for physical activity¤	3.59 [.619]	3.51, 3.67	3.46 [.681]	3.36, 3.57	1:1185/ 11.3	<.001
30. Physical activity is not necessary¤	3.79 [.495]	3.73, 3.85	3.67 [.573]	3.59, 3.76	1:1206/ 15.9	<.001
31. Physical activity is bad for me¤	3.77 [.530]	3.71, 3.84	3.71 [.541]	3.63, 3.79	1: 1213/ 3.5	.055
32. My friends and family disapprove of me being physically active¤	3.73 [.650]	3.65, 3.82	3.72 [.566]	3.64, 3.81	1:1165/.034	.826
**b. Survey responses about attitudes toward physical activity – domain “level of physical activity”**						
Sum score: level of physical activity	31.99 [6.322]	31.22, 32.77	28.40 [9.531]	27.09, 29.72	1:1306/ 66.7	<.001
33. I plan to be physically active	3.22 [.804]	3.12, 3.32	3.24 [.712]	3.14,3.35	1:1202/0.2	.617
34. My daily life is physically demanding	2.24 [.825]	2.14, 2.35	2.02 [.765]	1.90,2.13	1:1211/ 24.2	<.001
35. I don’t need to be physically active the family takes care of everything¤	3.73 [.548]	3.66, 3.80	3.55 [.619]	3.46,3.64	1:1226/ 27.3	<.001
36. My home situation requires that I am physically active	2.91 [.831]	2.80, 3.01	2.86 [.826]	2.74,2.98	1:1243/ 1.2	.284
37. I am physically active at least 150 minutes weakly	3.25 [.757]	3.16, 3.34	3.25 [.784]	3.14,3.37	1:1240/ 0.0	.922
38. I practice endurance training (walking/ running, Cycling, etc.)	2.50 [.968]	2.38, 2.62	2.73 [1.030]	2.58,2.88	1:1213/ 15.4	<.001
39. I practice muscle strengthening activities weekly	2.64 [.936]	2.52, 2.76	2.71 [.954]	2.57,2.85	1:1212/ 1.8	.179
40. I practice balance exercises	2.79 [.847]	2.68, 2.89	2.54 [.954]	2.40,2.68	1:1206/ 22.6	<.001
41. I cannot perform any physical activity¤	3.69 [.600]	3.62, 3.77	3.41 [.780]	3.30,3.53	1:1204/ 49.1	<.001
42. I have increased my level of physical activity after receiving the prosthesis	2.88 [.905]	2.77, 2.99	2.36 [1.019]	2.21,2.51	1:1214/ 89.3	<.001
43. I have reduced my level of physical activity after receiving the prosthesis¤	3.38 [.815]	3.28, 3.48	3.16 [.895]	3.03,3.29	1:1209/ 20.2	<.001
**c. Survey responses about attitudes toward physical activity – domain “function”.**						
Sum score: function.	14.19 [2.574]	13.88, 14.51	12.85 [3.562]	12.36, 13.34	1:1306/ 62.5	<.001
44. Physical activity is important for function of the operated leg	3.62 [.524]	3.56, 3.69	3.43 [.594]	3.35, 3.52	1:1258/ 36.8	<.001
45. Physical activity is good for the joints	3.64 [.525]	3.57, 3.70	3.44 [.594]	3.35, 3.52	1:1267/ 40.7	<.001
46. Physical activity is not necessary. the prosthesis alone gives me full function¤	3.58 [.672)	3.50, 3.66	3.33 [.750]	3.22, 3.43	1:1241/ 39.7	<.001
47. Physical activity is important for body function	3.64 [.574]	3.57, 3.71	3.51 [.592]	3.42, 3.59	1:1257/ 16.1	<.001
**d. Survey responses about attitudes toward physical activity – domain “kinesiophobia”.**						
Sum score: kinesiophobia	28.84 [4.407]	28.30, 29.38	26.76 [6.061]	25.93, 27.60	1:1306/ 51.3	<.001
48. I am afraid physical activity will be painful¤	3.46 [.688]	3.37, 3.54	3.26 [.690]	3.16, 3.36	1:1268/ 25.5	<.001
49. Physical activity may damage the prosthesis¤	3.53 [.609]	3.46, 3.61	3.25 [.650]	3.16, 3.34	1:1267/ 63.9	<.001
50. With a prosthesis vigorous physical activity should be avoided¤	3.75 [.523]	3.69, 3.81	3.16 [.729]	3.06, 3.26	1:1267/ 281.0	<.001
51. I will stop doing the physical activity if it hurts my operated leg¤	1.90 [.779]	1.80, 2.00	2.46 [.793]	2.35, 2.57	1:1254/ 157.7	<.001
52. I believe that my pain will always stop me from being physically active¤	3.25 [.735]	3.16, 3.34	3.26 [.677]	3.16, 3.35	1:1252/.039	.844
53. I will lead a normal lifestyle despite the pain/ prosthesis	3.32 [.730]	3.23, 3.41	3.09 [.735]	2.99, 3.20	1:1266/ 30.7	<.001
54. I am afraid of doing any physical activity without professional advice and guidance¤	3.36 [.762]	3.27, 3.45	3.19 [.731]	3.08, 3.29	1:1253/16.3	<.001
55. I feel confident knowing how to practice my physical activity	3.44 [.693]	3.35, 3.53	3.22 [.623]	3.13, 3.31	1:1272/ 34.8	<.001
56. I am coping well being physically active with my prothesis	3.40 [.693]	3.32, 3.49	3.23 [.715]	3.13, 3.33	1:1271/ 18.2	<.001

Means and standard deviations [SD]. Degrees of freedom (DF). F-statistics. Confidence Interval (CI) for the mean. Item numbers correspond to questionnaire (S1 File).

Likert scale 1–4. Higher score = more positive attitude toward physical activity.

¤ inverted score where the statements are negative toward physical activity.

^a^Score ranging from 0–36 (9 statements). Missing: range 1.5–10.5, mean 5.7%

^b^Score ranging from 0–44 (11 statements). Missing: range 5.1–8,2, mean 7.1%

^c^Score ranging from 0–16 (4 statements). Missing: range 3.3–5.3, mean 4.2%

^d^Score ranging from 0–36 (9 statements). Missing: range 2.9–4.4, mean 3.6%

### Explaining factors

Although many independent variables significantly explained the outcome of the survey, the explained variance was generally similar and low for both cohorts. The β-values in the regression models for pooled data for both cohorts showed that higher participation in sports was the strongest explaining factor for a more positive attitude toward PA in all four domains of the survey (Table 3), explaining 7.6% of *quality of life* (F = 92.5_1:1116_, p < .001), 8.1% of *physical activity* (F = 99.2_1:1116_, p < .001), 6.2% of *function* (F = 75.0_1:1116_, p < .001), and 4.6% of *kinesiophobia* (F = 54.6_1:1115_, p < .001). Older age was the strongest independent variable explaining a less positive attitude toward physical activity, explaining 3.7% of *quality of life* (F = 46.9_1:1185_, p < .001), 4.6% of *physical activity* (F = 58.1_1:1185_, p < .001), 2.9% of *function* (F = 37.9_1:1242_, p < .001), and 0.8% of *kinesiophobia* (F = 10.1_1:1161_, p < .001). Among health-related variables, more use of walking aids predicted a less positive attitude toward physical activity, explaining 5.3% of *quality of life* (F = 70.0_1:1245_, p < .001), 4.9% of *physical activity* (F = 65.3_1:1245_, p < .001), 2.8% of *function* (F = 37.3_1:1245_, p > .001), and 2.1% of *kinesiophobia* (F = 26.8_1:1190_, p < .001). For receiving service from the health system, participation in exercise classes predicted a more positive attitude toward PA explaining 4.5% of *quality of life* (F = 62.0_1:1306_, p < .001), while participation in prehab explained 2.4% of *physical activity* (F = 31.1_1:1245_, p < .001), and 1.0% of *function* (F = 14.0._1:1245_, p > .001). For *kinesiophobia,* longer time since surgery was the strongest positive explaining variable with 1.0% (F = 14.0_1:1237_, p < .001). Despite several additional variables being significant in all models, those added marginally explanatory value. Answers on the background questions as well as having hip or knee surgery did not significantly explained differences in survey outcomes. For the different cohorts, there were however some variations in the explanatory variance for additional variables (S4 Table).

**Table 3:** Stepwise regressions of grouped independent background variables explaining outcomes within each of the four dependent domains about attitudes toward physical activity for the pooled Norwegian and Dutch cohorts.

			ß	99.9%CI	p-value	R^2^
Quality of life	Demography	Age (years)	−.215	−1.926; −.733	<.001	
		Weight (kg)	−.105	−.443;.005	.001	
		Gender	−.086	−2.462;.233	.006	.046
	Lifestyle	Sports	.231	.818; 1.940	<.001	
		Education	.177	.513; 1.675	<.001	.105
	Health	Walking aids	−.227	−2.333; −.998	<.001	
		Prostheses (n)	−.081	−1.782; −.104	.003	.058
	Health service	Training (pre- & rehab (f))	.099	.340;.895	<.001	
		Information	.113	.169; 1.431	<.001	.056
Level physical activity	Demography	Age (years)	−.232	−2.485; −1.026	<.001	
		Weight (kg)	−.064	−.412;.082	.028	.049
	Lifestyle	Sports	.239	1.036; 2.459	<.001	
		Education	.122	.191; 1.675	<.001	
		Smoking	−.090	−1.781;.040	.002	
		Work	.079	−.097; 1.142	.005	.109
	Health	Walking aids	−.223	−2.840; −1.194	<.001	.049
	Health service	Prehab	.154	.389; 1.547	<.001	
		Information	.089	−.022; 1.447	.001	.031
Function	Demography	Age (years)	−.191	−.848; −.286	<.001	
		Weight (kg)	−.081	−.176;.014	.005	.034
	Lifestyle	Sports	.204	.310;.883	<.001	
		Education	.148	.150;.749	<.001	
		Smoking	−.070	−.636;.098	.016	.088
	Health	Walking aids	−.167	−.936;.271	<.001	
		Prostheses (n)	−.061	−.779; −.159	.030	.031
	Health service	Prehab	.104	.028;.480	<.001	
		Information	.062	−.095;.479	.028	.013
Kinesiofobia	Demography	Age (years)	−.107	−1.011; −.069	<.001	.011
	Lifestyle	Sports	.177	.364; 1.288	<.001	
		Education	.148	.237; 1.200	<.001	.065
	Health	Walking aids	−.150	−1.437; −.326	<.001	
		Knee^1^/ hip prost.^2^	.060	−.326; 1.434	.038	.024
	Health service	Surgery (t)	.106	.066; 1.060	<.001	.010

1 and 2 indicate code in the questionnaire for knee or hip prosthesis. (f) frequency, (t) time,

(n) number of consultations; last contact with physician or physiotherapist; (CI) Confidence Interval.

A positive beta value indicates a more positive attitude, while a negative value indicates a less positive attitude.

The explanatory strength for each model is noted in the R^2^ column for final model for each domain (demography, lifestyle, etc.) of significantly explaining background factors included in the models, nonsignificant variables removed. R^2^*100 = strength in percent.

## Discussion

The aim of this study was to gain insight into the attitude towards PA of Norwegian and Dutch patients 6–12 months after THA or TKA, investigating possible differences and explanatory factors that may exist between these countries. Overall, it can be concluded that patients from both countries have a generally positive attitude towards PA, with Norwegian patients having a more positive attitude compared to the Dutch patients. Among explaining factors, higher participation in sports was the strongest for a more positive attitude toward PA in all four domains of the survey: quality of life, level of PA, function, and kinesiophobia. Older age was strongest independent factor explaining a less positive attitude toward PA.

When looking at the survey domain “*quality of life*”, patients seem aware of the importance of PA in relation to health, fitness, and their social life and lifestyle, where most patients enjoyed being physically active. Most patients disagreed with the statement that PA is not necessary and bad for them. Compared to the Norwegian cohort, the Dutch cohort however scored lower on almost all questions within this category. The same was seen in the survey domain “*function*”, where participants from both cohorts showed awareness of the importance of PA in relation to function, but with a higher score of the Norwegian cohort. Age alone was the strongest background factor explaining a less positive attitude toward PA. For interpretation, it is important to note correlations between background factors (S2 Table); older age correlated with higher use of walking aids, less participation in sports, and lower level of education for both cohorts. Age may therefore in addition to being an explaining factor also be considered as a potential mediating factor where older participants are for instance more likely to use walking aids and may therefore score lower on being physically active which influences Likert scoring in the survey. The oldest participants are also less likely to have higher education as university degrees were less common for their generation, for example, the number of higher academic degrees in Norway has increased with 85%, and lower academic degrees with 76% since 1980 [24]. The same trend is seen in the Netherlands, with the percentage of higher educated people rising from 11.1% in 1981 to 35.5% in 2021, while the percentage of lower educated people dropped from 57.7% in 1981 to 25.8% in 2021 [25].

The background information revealed that participation in “prehab” was significantly higher in the Norwegian than in the Dutch cohort although relating to higher PA level for the latter, while higher training frequency explained higher PA level in the Norwegian cohort compared to the Dutch cohort where these were not related (S4 Table). These differences might be a result of the degree of participation in education about OA and the importance of a physically active lifestyle in relation to OA symptoms being standard care in the Norwegian OA intervention programs, such as AktivA [14]. In these programs, patients diagnosed with OA receive, next to standardized specific OA exercises, extensive education sessions, explaining and discussing in a group the importance of remaining physically active despite OA symptoms. From previous research in the Netherlands, it is known that the importance of remaining physically active is not always discussed with the patient by their orthopedic surgeon, although a survey among Dutch physiotherapists revealed that the vast majority of them said they were adhering to recommendations on postoperative training or exercise in THA and TKA, including patient education [21,26]. Education as part of pre- as well as postoperative treatment has as such the potential to increase the positive attitude of patients towards PA. Physiotherapy as such has an important role for enhancing PA; a systematic review of nineteen randomized control studies revealed that outpatient physical therapy performed in a clinic under the supervision of a trained physiotherapist may provide the best long-term outcomes after TKA [27]. A review about physical exercise after THA likewise provides convincing evidence for the effectiveness of individual interventions and regular exercise programs [28,29].

Although there are differences between the countries in the way health care is covered by insurance, most patients have access to physiotherapy and consequently could be educated by physiotherapists about the advantages of being physically active. In the Netherlands, health insurance is provided by private companies and is mandatory for all citizens. As a result, Dutch citizens have a free choice out of multiple insurance companies [30]. These companies offer a basic health insurance package of which the content is established by the Dutch government. In addition, people can choose additional health insurance packages of which the content can vary between different insurance companies. The first 12 sessions of supervised exercise therapy for people with hip or knee OA are covered by the basic health insurance, as are the THA and TKA surgeries itself. For postoperative physiotherapy however additional coverage is needed [31]. In practice, most people in need of an arthroplasty have additional coverage where 92.7% with THA and 96.9% with TKA had postoperative physiotherapy in a primary care practice or at home, which would give ample opportunity to educate patients about the advantages of being physically active preoperatively and postoperatively [32]. In contrast to the Netherlands, Norway has a fully state-funded healthcare system, where citizens pay a fee for healthcare from the public health services, which includes physiotherapists. This means that patients can choose a physiotherapist both before and after surgery with minimal financial costs. Some of the residents have additional private health insurance which ensures they have quick access to a physiotherapist. THA and TKA are included in this system, and very few use private insurance for such surgeries. The scope and volume of pre- and post-operative physiotherapy is roughly the same as in the Netherlands.

Regarding the second domain ‘level of PA’, both Norwegian and Dutch participants had a positive attitude, but again the Norwegian cohort showed a more positive overall attitude. Norwegian participants considered their daily life more physically demanding than the Dutch and more Norwegian participants indicated that they have increased their PA after surgery and performed balance training. A higher number of the Dutch participants indicated that they practice endurance training. In the Netherlands, bicycling is a common means of getting around. Notably, in the survey (S1 File), bicycling to work was given as an example of PA. Although the same number of participants in both cohorts indicated to comply with the 150-minutes per week PA guideline, geographical and cultural differences could explain (part of) these differences [33]. In addition, geographical, infra structural, and seasonal differences play a role, Norway being mountainous, more rural, and having long winters with snow compared to the Netherlands being flat, urban and having winters without snow may explain the types of PA.

A positive attitude towards PA appeared to be mainly explained by a high participation in sports. Not surprisingly, being physically active has a positive effect on the attitude towards it. This finding implicates that it is of utmost importance to guide patients to become active again after hip or knee replacement. As older age appeared to be related to a less positive attitude towards PA, this is especially important in the older population. However, since the explaining values were generally very low for each separate domain of factors, it can be concluded that attitude is influenced by many different factors. Moreover, as a cross-sectional study, no definite conclusions can be drawn about causality; it is just as likely that people with a positive attitude towards PA are more active as the other way round.

The attitude toward PA may be negatively influenced by kinesiophobia, but scores indicated that fear of pain did not prevent the participants in the survey from being physically active. The Norwegian cohort scored significantly higher on all but one item within this category “*I will stop doing the PA if it hurts my operated leg*”. Both cohorts scored however low on this statement which may reflect fear or maybe a healthy caution, depending on the interpretation of the respondent. Thus, a low score here is ambiguous and does not necessarily mean a negative attitude to PA.

To the best of our knowledge, this is the first survey investigating the general attitude towards PA of patients after hip or knee replacement. The strength of our study is the transnational catchment area and the relatively high number of participants. We also consider the extensive background information, and the variety of questions intended to determine attitudes relevant as discussed in the international PAIR study group and validated by patients in pilot studies across countries. Previous studies have focused on attitudes toward PA, investigating perceptions of facilitators and barriers for PA using interviews [12], while other studies have focused on the attitudes of health care professionals towards PA and on the attitude of patients towards specific (exercise) interventions [34–36].

Our study however also has some limitations. First, the recruitment of participants differed between countries. While in Norway, patients were recruited representatively throughout the whole country by NAR, recruitment in the Netherlands was limited to the patients from four participating hospitals, although geographically spread across the country. Second, there could be a selection bias, as it is possible that those patients who have a positive attitude towards PA were more likely to participate in the survey, as such increasing the score on the survey. Still, the domain in the survey with the highest number of missing data was the part about physical activity. With self-administered surveys there is also a risk of reporting bias where respondents tend to answer questions in accordance with normative values [37]. In our survey most participants claimed that they follow WHO’s recommendation of 150 minutes of PA a week. Considering studies using a device-based measurement method, e.g., accelerometry, middle aged Europeans are far away from meeting the WHO recommendation [38]. It is thus likely that participants overestimated time spent on PA. As this would be the case in both cohorts it did not hamper the comparison between countries. Third, the participants may have interpreted some statements differently. Even though the survey was piloted for content and construct validity, it is still possible that individual interpretations differed. Finally, as weight and height were answered in categories, body mass index (BMI) could not be calculated and used in the regression analyses.

## Conclusion

In conclusion, patients seem to have an overall positive attitude towards PA after hip or knee replacement. Norwegian patients have a slightly more positive attitude compared to Dutch patients, which might be the result of more formalized and extensive education of the benefits of PA for patients in Norway along with participation in OA school and prehab. Older age alone explained a less positive attitude toward PA and correlated with factors related to a negative attitude toward PA such as use of walking aids, lower education, and less engagement in sports. Our results point out the importance of encouraging also elderly patients to engage in physical activity and provide individual guidance with consideration of needs and limitations.

## Supporting information

S1 File PAIR questionnaire. (PDF)

S2 Table Correlations between background factors, based on Pearson. (DOCX)

S3 Table Independent background variables by group. (DOCX)

S4 Table Stepwise regressions of grouped independent background variables explaining outcomes within each of the four dependent domains about attitudes toward physical activity for Norwegian (NO) and Dutch (NL) cohorts. (DOCX)
