# Supplementary material for: Attitude toward physical activity after total hip or knee replacement: A cross-sectional survey study of Dutch and Norwegian patients
Source: PLoS One. 2026 Jan 23;21(1):e0325746. doi: 10.1371/journal.pone.0325746 (PMC12829782; doi:10.1371/journal.pone.0325746)
Supplement: S1 File — (PDF) [file pone.0325746.s001.pdf]

Questionnaire ID number:………………

PAIR P: for Patients after Arthroplasty Surgery

You have received this questionnaire because you have had a hip or knee replacement. We ask you about your attitude towards physical activity. Physical activity: tasks increasing energy consumption above resting level, such as *walking, gardening, biking to work, includes exercise, sports and more.*

We kindly ask you to fill out part 2-4. Please cross one alternative.

**1**	BACKGROUND SETTING For administrative use only				
1	**Region and country:**				
**2**	**Area**	[1] Rural ☐	[2] Suburban ☐	[3] Urban ☐	
**3**	**Type of clinic**	[1] Orthopaedic clinic ☐	[2] Hospital policlinic ☐	[3] Community health clinic ☐	[4] Physiotherapy clinic ☐
		[5] Health Studio ☐	[6] Rehab clinic ☐		
**4**	**Distributor**	[1] General Physician ☐	[2] Rehabilitation Physician ☐	[3] Orthopaedic surgeon ☐	[4] Physiotherapist ☐
		[5] Nurse	[6] Occupational therapist ☐	[7] Medical student ☐	[8] Exercise trainer (non-medical) ☐
**2**	PATIENT INFORMATION				
	Response alternatives may not exactly match your situation. Chose the alternative closest to your situation. IF NOTHING ELSE IS SPECIFIED YOU WILL MARK *ONLY ONE ALTERNATIVE!!!* PLEASE ANSWER ALL QUESTIONS!				
5	**Gender**	[1] Male ☐	[2] Female ☐	[3] Other ☐	☐
6	**Age (years)**	< 30 ☐	31-40 ☐	41-50 ☐	51-60 ☐
		61-70 ☐	71-80 ☐	81-90 ☐	> 90 ☐
7	**Height (cm)**	< 150 ☐	150-160 ☐	161-170 ☐	171-180 ☐
		181-190 ☐	>190 ☐		
8	**Weight (kg)**	< 45 ☐	46-50 ☐	51-55 ☐	56-60 ☐
		61-65 ☐	66-70 ☐	71-75 ☐	76-80 ☐
		81-85 ☐	86-90 ☐	91-95 ☐	96-100 ☐
		101-105 ☐	106-110 ☐	111-115 ☐	> 115 ☐
9	**Education Level**	[1] Grammar school ☐	[2] College ☐	[3] University graduate ☐	[4] University post-graduate ☐

10	**Occupation sector (if on sick leave or between jobs, please check occupation**	[1] Domestic / at home ☐	[2] Office / mostly sitting ☐	[3] Lighter work / walking and standing ☐	[4] Heavy physically demanding work ☐
11	**Living situation**	[1] Single ☐	[2] Married / living together ☐		
12	**Smoking**	[1] Never ☐	[2] Have stopped ☐	[3] Less than 10 /day ☐	[4] More than 10 / day ☐
13	**Prior sports participation level**	[1] None ☐	[2] Leisure / irregular ☐	[3] Moderate / regular ☐	[4] High / competitive ☐
14	**Walking aids**	[1] None ☐	[2] Cane ☐	[3] Crutches ☐	[4] Roller ☐
15	**Present surgery**	[1] Knee ☐	[2] Hip ☐		
	Question 16 and 17: here you can choose several alternatives. If so, please also mark the alternative **“several”**				
16	**Prosthesis <u>earlier</u>**	[1] One knee ☐<br>[5] Both knees ☐	[2] One hip ☐<br>[6] Both hips ☐	[3] Shoulder ☐<br>[7] Ankle ☐	[4] None ☐<br>[8] **Several** ☐
17	**Other diagnosis**	[1] Heart ☐<br>[5] Diabetic ☐<br>[8] Psychiatric ☐	[2] Rheumatic ☐<br>[6] Respiratory ☐<br>[9] Geriatric ☐	[3] Neurologic ☐<br>[7] Born with anatomic anomaly ☐	[4] None ☐<br>[10] **Several** ☐
3	INFORMATION FROM HEALTH SERVICE				
	Response alternatives may not exactly match your situation. Chose the alternative closest to your situation. IF NOTHING ELSE IS SPECIFIED YOU WILL MARK *ONLY ONE ALTERNATIVE!!!*<br>PLEASE ANSWER ALL QUESTIONS!				
18	**How long ago since surgery**	[1] < 1 week ☐<br>[5] 3-6 months ☐	[2] 1 week–1 month ☐<br>[6] > 6 months ☐	[3] 1-2 months ☐	[4] 2-3 months ☐
19	**How long ago since last contact with health personnel after surgery**	[1] < 1 week ☐<br>[5] 3-6 months ☐	[2] 1 week–1 month ☐<br>[6] > 6 months ☐	[3] 1-2 months ☐	[4] 2-3 months ☐

20	**I participated in a prehab program (exercise program before surgery)**	1 None ☐	2 Weekly < 1 month ☐	3 Weekly for 1-2 months ☐	4 Weekly > 2 months ☐
21	**I participated in a rehab program (exercise program after surgery)**	1 None ☐	2 Weekly <1 month ☐	3 Weekly for 1-2 months ☐	4 Weekly > 2 months ☐
	Question 22 and 23: here you can choose several alternatives! If so, please also mark the alternative **"several"**				
22	**Information about importance of physical activity was given by**	1 none ☐<br>5 physio therapist ☐	2 physician ☐<br>6 exercise trainer ☐	3 secretary ☐<br>7 occupational therapist ☐	4 nurse ☐<br>8 **Several** ☐
23	**Information about importance of physical activity was given**	1 None ☐	2 Orally ☐	3 Written ☐	4 **Several** ☐
24	**Advice and supervision were individually personalized**	1 No ☐	2 Yes ☐		
4	ATTITUDES TOWARD PHYSICAL ACTIVITY				

Physical activity: tasks increasing energy consumption above resting level, such as *walking, gardening, biking to work, includes exercise, sports and more.*

Rate each statement by placing a mark at ONE choice closest to your opinion!

PLEASE ANSWER ALL QUESTIONS!

4a	Quality of life				
		Strongly disagree	***Disagree***	***Agree***	***Strongly agree***
25	**Physical activity is important for my fitness**	☐	☐	☐	☐
26	**Physical activity is important for my health**	☐	☐	☐	☐

27	**Physical activity is important for my social life and life- style**	☐	☐	☐	☐
28	**I enjoy being physically active**	☐	☐	☐	☐
29	**I do not have time for physical activity**	☐	☐	☐	☐
30	**Physical activity is not necessary**	☐	☐	☐	☐
31	**Physical activity is bad for me**	☐	☐	☐	☐
32	**My friends and family disapprove of me being physically active**	☐	☐	☐	☐
**4b**	Level of physical activity				
		Strongly disagree	***Disagree***	***Agree***	***Strongly agree***
33	**I plan to be physically active**	☐	☐	☐	☐
34	**My daily life is physically demanding**	☐	☐	☐	☐
35	**I don't need to be physically active the family takes care of everything**	☐	☐	☐	☐
36	**My home situation requires that I am physically active**	☐	☐	☐	☐

37	I am physically active at least 150 minutes weakly	☐	☐	☐	☐
38	I practice endurance training (walking/ running, cycling, etc)	☐	☐	☐	☐
39	I practice muscle strengthening activities weekly	☐	☐	☐	☐
40	I practice balance exercises	☐	☐	☐	☐
41	I cannot perform any physical activity	☐	☐	☐	☐
42	I have increased my level of physical activity after receiving the prosthesis	☐	☐	☐	☐
43	I have reduced my level of physical activity after receiving the prosthesis	☐	☐	☐	☐
4c	Function				
		Strongly disagree	*Disagree*	*Agree*	*Strongly agree*
44	Physical activity is important for function of the operated leg	☐	☐	☐	☐
45	Physical activity is good for the joints	☐	☐	☐	☐
46	Physical activity is not necessary, the prosthesis	☐	☐	☐	☐

	alone gives me full function				
47	Physical activity is important for body function	☐	☐	☐	☐
4d	Fear of Movement				
		Strongly disagree	*Disagree*	*Agree*	*Strongly agree*
48	I am afraid physical activity will be painful	☐	☐	☐	☐
49	Physical activity may damage the prosthesis	☐	☐	☐	☐
50	With a prosthesis vigorous physical activity should be avoided	☐	☐	☐	☐
51	I will stop doing the physical activity if it hurts my operated leg	☐	☐	☐	☐
52	I believe that my pain will always stop me from being physically active	☐	☐	☐	☐
53	I will lead a normal lifestyle despite the pain / prosthesis	☐	☐	☐	☐
54	I am afraid of doing any physical activity without professional advice and guidance	☐	☐	☐	☐

55	**I feel confident knowing how to practice my physical activity**	☐	☐	☐	☐
56	**I am coping well being physically active with my prothesis**	☐	☐	☐	☐
