# Supplementary material for: Attitude toward physical activity after total hip or knee replacement: A cross-sectional survey study of Dutch and Norwegian patients
Source: PLoS One. 2026 Jan 23;21(1):e0325746. doi: 10.1371/journal.pone.0325746 (PMC12829782; doi:10.1371/journal.pone.0325746)
Supplement: S2 Table — (DOCX) [file pone.0325746.s002.docx]

**S2 Table. Correlations between background factors, based on Pearson.**

|  | Norway: p/ R* | The Netherlands: p /R* | All: p/ R* |
| --- | --- | --- | --- |
| Walking aids |  |  |  |
| Sports | .098 / -.064 | .037 /-.091 | <.001 / -.099 |
| Smoking | - | - | - |
| Marriage status | .022 / -.087 | <.001 / -.201 | <.001 7 -.149 |
| Work | .448 / -.029 | .013 / -.111 | .066 / -.054 |
| Education | .094 / -.065 | .178 / -.060 | <.001 / -.111 |
| Weight | - | - | - |
| Age | <.001 / .129 | <.001 / .264 | <.001 / .206 |
| Gender | 0.37 / .082 | .002 / .133 | <.001 / .109 |
| Country | n/a | n/a | <.001 / .171 |
| Information | - | - | - |
| Participation in training | - | - | - |
| Rehab | .210 /.048 | .004 /.129 | .005 /.080 |
| Prehab | - | - | - |
| Diagnoses | .005 / .107 | .081 / 0.74 | .002 / .087 |
| Knee / hip prosthesis | .121 / .059 | .607 /.022 | .070 / .051 |
| Sports |  |  |  |
| Smoking | .002 / -.119 | .082/ -.076 | <.001 /-.109 |
| Marriage status | - | - | - |
| Work | - | - | - |
| Education | <.001 / .215 | <.001 / .287 | <.001 / .268 |
| Weight | - | - | - |
| Age | .013 / -.096 | .010 / -.111 | <.001 / -.102 |
| Gender | <.001 / -.222 | .878 / .007 | <.001 / -.123 |
| Country | n/a | n/a | <.001 / -.139 |
| Information | - | - | - |
| Participation in training | .928 / -.003 | <.001 / .165 | .006 / .078 |
| Rehab | .533 / -.024 | .049 / .086 | .402 / -.024 |
| Prehab | .675 / .016 | <.001 / .158 | .002 / .087 |
| Diagnoses | - | - |  |
| Knee / hip prosthesis | - | - | - |
| Smoking |  |  |  |
| Marriage status | - | - | - |
| Work | - | - | - |
| Education | - | - | - |
| Weight | .013 / .095 | .042 / .088 | .002 / .089 |
| Age | - | - | - |
| Gender | .054 / -.075 | .009 / -.113 | .002 / -.091 |
| Country | n/a | n/a | .038 / .058 |
| Information | - | - | - |
| Participation in training | - | - | - |
| Rehab | - | - | - |
| Prehab | - | - | - |
| Diagnoses | - | - | - |
| Knee / hip prosthesis | - | - | - |
| Marriage Status |  |  |  |
| Work | .056 / .072 | .164 / .062 | .019 / .068 |
| Education | - | - | - |
| Weight | - | - | - |
| Age | .533 / -.024 | <.001 / -.150 | .003 / -.085 |
| Gender | .001 / -.125 | 0.52 / .053 | <.001 / -.106 |
| Country | n/a | n/a | - |
| Information | - | - | - |
| Participation in training | .164 / .052 | .079 / .074 | .023 / .064 |
| Rehab | - | - | - |
| Prehab | - | - | - |
| Diagnoses | - | - | - |
| Knee / hip prosthesis | - | - | - |
| Work |  |  |  |
| Education | .036 / -.080 | .002 / .142 | .589 / .017 |
| Weight | .007 / .102 | .005 / .124 | <.001 / .112 |
| Age | <.001 / -.238 | .001 / -.284 | <.001 / -.249 |
| Gender | .431 / -.031 | <.001 / -.322 | <.001 / -.166 |
| Country | n/a | n/a | - |
| Information | - | - | - |
| Participation in training | .021 / .078 | .537 / .027 | .018 / .067 |
| Rehab | .005 / .107 | .300 / .046 | .005 / .081 |
| Prehab | - | - | - |
| Diagnoses | - | - | - |
| Knee / hip prosthesis | - | - | - |
| Education |  |  |  |
| Weight | - | - | - |
| Age | <.001 -/ -.149 | .013 / -.109 | <.001 / -.116 |
| Gender | - | - | - |
| Country | n/a | n/a | <.001 / -.350 |
| information | .019 / .091 | .014 / .107 | .035 / .080 |
| Participation in training | .979 / -.001 | .539 / .027 | .035 / .060 |
| Rehab | - | - | - |
| Prehab | .831 / .009 | .981 / .001 | .005 / .81 |
| Diagnoses | - | - | - |
| Knee / hip prosthesis | - | - | - |
| Weight |  |  |  |
| Age | <.001 / -.265 | <.001 / -.209 | <.001 / -.236 |
| ~~Gender~~ |  | n/a men are heavier |  |
| Country | n/a | n/a | - |
| Information | .719 / .013 | .012 / .105 | .046 / .056 |
| Participation in training | - | - | - |
| Rehab | - | - | - |
| Prehab | - | - | - |
| Diagnoses | - | - | - |
| Knee / hip prosthesis | .019 / .089 | .166 / -.058 | .740 / .009 |
| Age |  |  |  |
| Gender | .786 / .011 | .027 / .093 | .072 / .052 |
| ~~Country~~ | n/a | n/a | - |
| Information | <.001 / -.175 | <.001 / -.184 | <.001 / -.178 |
| Participation in training | .009 / -.099 | .030 / -.091 | <.001 / -.095 |
| Rehab | .010 / -.099 | .401 / -.036 | .016 / -.089 |
| Prehab | - | - | - |
| Diagnoses | - | - | - |
| Knee / hip prosthesis | - | - | - |
| Gender |  |  |  |
| Country | n/a | n/a | - |
| Information | - | - | - |
| Participation in training | <.001 / .185 | .292 / .044 | <.001 / .124 |
| Rehab | <.001 / .205 | .565 / .025 | <.001 / .101 |
| Prehab | <.001 / .129 | .120 / .067 | <.001 / .101 |
| Diagnoses | - | - | - |
| Knee / hip prosthesis | - | - | - |
| Country |  |  |  |
| Information | n/a | n/a | .008 / .073 |
| Participation in training | n/a | n/a | <.001 / -.157 |
| Rehab | n/a | n/a | - |
| Prehab | n/a | n/a | <.001 /-.218 |
| Diagnoses | n/a | n/a | - |
| Knee / hip prosthesis |  |  | .002 / .085 |
| Information |  |  |  |
| Participation in training | .001 / .119 | <.001 / .183 | <.001 / .125 |
| Rehab | .043 / .076 | .002 / .134 | <.001 / .100 |
| Prehab | .050 / .074 | .960 / .002 | .395 / .024 |
| Diagnoses | .128 / .057 | .023 / .095 | .005 / .079 |
| Knee / hip prosthesis | - | - | - |
| Participation in training |  |  |  |
| Rehab | <.001 / .835 | <.001 / .985 | <.001 / .837 |
| Prehab | <.001 / .830 | <.001 / .704 | <.001 / .795 |
| Diagnoses | - | - | - |
| Knee / hip prosthesis | - | - | - |
| Rehab |  |  |  |
| Prehab | <.001 / .413 | <.001 / .304 | <.001 / .370 |
| Diagnoses | - | - | - |
| Knee / hip prosthesis | - | - | - |
| Prehab |  |  |  |
| Diagnoses | - | - | - |
| Knee / hip prosthesis | - | - | - |
| Diagnoses |  |  |  |
| Knee / hip prosthesis | - | - | - |

* p = p-value, R = correlation coefficient
