# Supplementary material for: Attitude toward physical activity after total hip or knee replacement: A cross-sectional survey study of Dutch and Norwegian patients
Source: PLoS One. 2026 Jan 23;21(1):e0325746. doi: 10.1371/journal.pone.0325746 (PMC12829782; doi:10.1371/journal.pone.0325746)
Supplement: S3 Table — (DOCX) [file pone.0325746.s003.docx]

| **S3 Table. Independent background variables by group** | |
| --- | --- |
| Demography | Age, gender, marriage status, body weight** |
| Lifestyle | Education, work, smoking, sports |
| Health | Walking aids, knee / hip prosthesis, other prostheses*, diagnoses* |
| Health service | prehab/ rehab*, information from health service on physical activity*, time since prosthesis surgery, time since last consultation with physiotherapist / orthopedist |

*****sum scores: number of prostheses, number of diagnoses, frequency of participation in prehab / rehab, number of information sources about physical activity
**estimated body weight v/ score (f ex 5 = 61-65 kg, 6 = 66-70 kg, etc)
