# Supplementary material for: Attitude toward physical activity after total hip or knee replacement: A cross-sectional survey study of Dutch and Norwegian patients
Source: PLoS One. 2026 Jan 23;21(1):e0325746. doi: 10.1371/journal.pone.0325746 (PMC12829782; doi:10.1371/journal.pone.0325746)
Supplement: S4 Table — (DOCX) [file pone.0325746.s004.docx]

| **S4 Table. Stepwise regressions of grouped independent background variables explaining outcomes within each of the four dependent domains about attitudes toward physical activity for Norwegian (NO) and Dutch (NL) cohorts.** | | | | | | | | | | |
| --- | --- | --- | --- | --- | --- | --- | --- | --- | --- | --- |
|  | | | ß | | 99.9%CI | | p-value | | R^2^ | |
|  |  |  | NO | NL | NO | NL | NO | NL | NO | NL |
| Quality of life | Demography | Weight (kg) | -.179 | excl. | -.368; -.060 | n/s | <.001 | n/s | - | n/s |
|  |  | Age (years) | -.149 | -.247 | -1.093; -.106 | -2.733; -.780 | <.001 | <.001 | .039 | .059 |
|  | Lifestyle | Sports | .168 | .303 | .167; 1.148 | 1.212; 3.492 | <.001 | <.001 | - | - |
|  |  | Education | .144 | .107 | .074; 1.094 | -.378; 2.381 | <.001 | .017 | .057 | .118 |
|  | Health | Walking aids  Prostheses (n) | -.231  n/s | -.179  -.086 | -2.140; -.646  n/s | -2.451; -.310  -2.563; .594 | <.001  n/s | <.001  .040 | .052  n/s | -  .037 |
|  | Health service | Information | .094 | .145 | -.129; .936 | .003; 2.003 | .012 | <.001 | - | - |
|  |  | Surgery (t) | .115 | excl. | -.039; .977 | n/s | .002 | n/s | .018 | n/s |
|  |  | Consultation (t) | excl. | -.143 | n/s | -1.044; .003 | n/s | .001 | n/s | .040 |
| Level physical activity | Demography | Weight (kg) | -.104 | excl. | -.451; -.049 | n/s | .008 | n/s | - | n/s |
|  |  | Age (years) | -.191 | -.252 | -1.982; -.382 | -3.264; -.962 | <.001 | <.001 | .034 | .062 |
|  | Lifestyle | Sports | .189 | .308 | .384; .1.954 | 1.521; 4.114 | <.001 | <.001 | - | - |
|  |  | Education | .101 | excl. | -.170; 1.468 | n/s | .009 | n/s | - | - |
|  |  | Work | .086 | n/s | -.230; 1.242 | n/s | .023 | n/s | .058 |  |
|  |  | Smoking | excl. | -.105 | n/s | -2.781; .418 | n/s | .015 | n/s | .108 |
|  | Health | Walking aids | -.172 | -.206 | -2.696; -.438 | -3.051; -.604 | <.001 | <.001 | .028 | .041 |
|  | Health service | Information | .099 | .152 | -.169; 1.557 | .065; 2.374 | .008 | <.001 | - | - |
|  |  | Prehab  Training (pre- & rehab (f))  Consultation (t) | excl.  .147  n/s | .127  n/s  -.098 | n/s  .066; .733  n/s | -.144; 2.412  n/s  -1.015; .194 | n/s  <.001  n/s | .003  n/s  .025 | n/s  .032  n/s | n/s  -  .044 |
| Function | Demography | Weight (kg) | -.109 | excl. | -.199; .031 | n/s | .016 | n/s | - | n/s |
|  |  | Age (years) | -.152 | -.186 | -.701; -.040 | -1.021; -.146 | <.001 | <.001 | - | .033 |
|  |  | Gender | .095 | excl. | -.232; 1.150 | n/s | .029 | n/s | .039 | - |
|  | Lifestyle | Sports | .163 | .257 | .090; .733 | .386; 1.452 | <.001 | <.001 | - | - |
|  |  | Education | .102 | .102 | .060; .613 | -.203; 1.088 | .007 | .024 | - | .088 |
|  |  | Smoking | -.086 | excl. | -.733; .138 | n/s | .024 | n/s | .054 | - |
|  | Health | Walking aids | -.090 | -.166 | -.812; .133 | -1.051; -.093 | .018 | <.001 | .007 | - |
|  |  | Prostheses (n) | excl. | -.106 | n/s | -1.245; .168 | n/s | .012 | n/s | .036 |
|  | Health service | Information | excl. | .115 | n/s | -.096; .815 | n/s | .009 | n/s | - |
|  |  | Consultation (t) | excl. | -.120 | n/s | -.436; .041 | n/s | .006 | n/s | .025 |
| Kinesiofobia | Demography | Weight (kg) | -.087 | excl. | -.300; .060 | n/s | .028 | n/s | - | n/s |
|  |  | Age (years)  Married / living together | -.144  n/s | excl.  .108 | -1.214; -.061  n/s | n/s  -.390; 2.971 | <.001  n/s | <.001  .011 | .019  n/s | n/s  .010 |
|  | Lifestyle | Sports | .130 | .246 | .009; 1.083 | .528; 2.057 | <.001 | <.001 | - | .058 |
|  |  | Education | .114 | excl. | -.065; 1.060 | n/s | .004 | n/s | - | n/s |
|  |  | Smoking | -.093 | excl. | -1.263; .192 | n/s | .015 | n/s | .047 | n/s |
|  | Health | Walking aids | -.214 | -.178 | -2.243; -.602 | -1.702; -.210 | <.001 | <.001 | - | - |
|  |  | Diagnoses (n) | excl. | .085 | n/s | -.510; 2.102 | n/s | .044 | - | .033 |
|  |  | Knee^1^ / hip prost.^2^ | .074 | excl. | -.429 ;1.691 | n/s | .049 | n/s | .049 | n/s |
|  | Health service | Surgery (t)  Consultation (t) | excl.  .099 | .103  excl. | n/s  -.059; .501 | -.234; 1.349  n/s | n/s  .009 | .020  n/s | n/s  .008 | .009  n/s |
| 1 and 2 indicate code in the questionnaire for knee or hip prosthesis. (f) frequency, (t) = time, (n) number of consultation; last contact with physician or physiotherapist, (CI) Confidence Interval. | | | | | | | | | | |
| A positive beta value indicates a more positive attitude, while a negative value indicates a less positive attitude. R^2^*100 indicates the explanatory strength per group of independent variables (in percentage). Excluded variables that are denominated as non-significant n/s, are not included in the strength calculation. A dash indicates that the variable is included in the strength calculation. | | | | | | | | | | |
